# Current clinical practice gaps in the treatment of intermediate- and high-risk non-muscleinvasive bladder cancer (NMIBC) with emphasis on the use of bacillus Calmette- Guérin (BCG): results of an international individual patient data survey (IPDS)

**DOI:** 10.1111/bju.12012

**Published:** 2013-03-01

**Authors:** J Alfred Witjes, Joan Palou, Mark Soloway, Donald Lamm, Ashish M Kamat, Maurizio Brausi, Raj Persad, Roger Buckley, Marc Colombel, Andreas Böhle

**Affiliations:** 1 Department of Urology, Radboud University Nijmegen Medical CentreNijmegen, The Netherlands; 2 Department of Urology, Fundació Puigvert, Universitat Autònoma de BarcelonaBarcelona, Spain; 3 Department of Urology, University of Miami School of MedicineMiami, FL, USA; 4 Department of Surgery, University of Arizona, BCG OncologyPhoenix, AZ, USA; 5 Department of Urology, MD Anderson Cancer CentreHouston, TX, USA; 6 Department of Urology, AUSL ModenaModena, Italy; 7 Department of Urology/Surgery, Bristol Royal Infirmary and Bristol Urological InstituteBristol, UK; 8 Department of Urology, North York General HospitalToronto, ON, Canada; 9 Department of Urology, Claude Bernard University, Hôpital Edouard HerriotLyon, France; 10 Department of Urology, HELIOS Agnes Karll HospitalBad Schwartau, Germany

**Keywords:** bacillus Calmette-Guérin (BCG), guideline adherence, non-muscle-invasive bladder cancer (NMIBC), practice gaps, physician survey

## Abstract

**Objectives:**

- To examine the management of intermediate- and high-risk non-muscle-invasive bladder cancer (NMIBC), particularly with regard to the use of bacillus Calmette-Guérin (BCG) therapy, in North America and Europe.
- To compare NMIBC management practices to European Association of Urology (EAU) and American Urological Association (AUA) guideline recommendations for the management of intermediate- and high-risk NMIBC.

**Patients and Methods:**

- In all, 102 urologists from Europe and North America participated in this retrospective on-line chart review, which was conducted between 1 April 2011 and 30 April 2012.
- Participants selected the charts of the first 10 intermediate- (defined as multiple or recurrent low-grade tumours) or high-risk (defined as any T1 and/or high-grade/G3 tumours and/or carcinoma *in situ*) patients who underwent transurethral resection of bladder tumour in 2009.
- Physicians retrospectively reviewed the charts and completed an on-line survey consisting of questions related to diagnosis, planned treatment, treatment status and follow-up.
- In all, 971 patients (197 intermediate-risk; 774 high-risk) were included in the analysis; frequency counts and associated percentages were used to analyse treatment variables.

**Results:**

- In all, 47% of intermediate-risk patients received EAU or AUA guideline-recommended intravesical therapy: intravesical chemotherapy, BCG induction therapy or BCG induction plus maintenance.
- Of the high-risk patients, 50% received maintenance BCG as recommended by the EAU and the AUA; although not recommended for high-risk NMIBC, 12.5% received intravesical chemotherapy.
- Of patients prescribed maintenance BCG, 93% were scheduled for at least 1 year of therapy.
- Notably, only 15% discontinued BCG maintenance and, of these discontinuations, 65% were due to reasons unrelated to BCG-associated adverse events.

**Conclusions:**

- There is significant non-adherence to EAU and AUA guideline recommendations for BCG use in intermediate- and high-risk NMIBC.
- However, most of those patients prescribed BCG maintenance therapy are scheduled for at least 1 year of therapy, as recommended by current guidelines for NMIBC management, and BCG maintenance discontinuation is low.

## Introduction

Non-muscle-invasive bladder cancer (NMIBC), i.e. Ta/T1/carcinoma *in situ* (CIS), represents a heterogeneous group of tumours with varying oncological outcomes. Therefore, risk stratification is imperative for determining the appropriate management strategy based on the patient's risk of recurrence and progression. Although the European Organisation for Research and Treatment of Cancer (EORTC) risk tables are recommended for estimating this risk [1,2], the use of these tables is not always practical. Furthermore, recent evidence suggests that these tables tend to overestimate both the risk of recurrence and progression in patients with T1G3 tumours treated with BCG [3]. Recently, the International Bladder Cancer Group (IBCG) proposed the following more practical definitions of risk based on review of current clinical practice guidelines for NMIBC [4]:

- *Low risk:* solitary, primary low-grade (Ta) tumour (these tumours are at low risk of recurrence and progression)
- *Intermediate risk:* multiple or recurrent low-grade tumours (these tumours are at intermediate to high risk of recurrence, but low to intermediate risk of progression)
- *High risk:* any T1 and/or high-grade/G3 and/or CIS (these tumours are at high risk of recurrence and progression, with progression being the primary concern)

Given the risks of recurrence, progression and mortality in high-risk disease, timely recognition and management of NMIBC is imperative. In 2008, the European Association of Urology (EAU) NMIBC guidelines recommended BCG with ≥1 year of maintenance as the ‘gold-standard’ intravesical treatment for high-risk patients. For intermediate-risk NMIBC, BCG with ≥1 year of maintenance or intravesical chemotherapy were recommended (Table 1) [5]. These guidelines have essentially remained unchanged since 2008 [2]. The most recent AUA guidelines, which were published in 2007 (and reviewed to confirm validity in 2010), recommend an induction course of BCG or mitomycin C (MMC) for patients at high-risk of recurrence but low risk of progression (intermediate-risk) [6,7]. Although the AUA acknowledges that maintenance therapy is more effective in reducing recurrences than induction therapy alone, routine maintenance is considered optional in patients with intermediate-risk disease given that the side-effects and costs associated with treatment may outweigh the benefits in these patients. Similar to the EAU guidelines, the AUA recommends BCG induction followed by maintenance as the intravesical treatment of choice for high-risk NMIBC [6,7] (Table 1).

**Table 1:** The 2008 EAU and 2007 AUA guidelines for the management of NMIBC [5–7]

Risk category	EAU recommendations	AUA recommendations
Low risk	TURBT Single, immediate postoperative chemotherapeutic instillation (grade A)	TURBT (standard) Single, immediate postoperative chemotherapeutic instillation (recommendation)
Intermediate risk	TURBT Single, immediate postoperative chemotherapeutic instillation followed by: – Further instillations of chemotherapy (grade A) for 6–12 months (grade B), or – BCG with a minimum of 1 year of maintenance (grade A)	TURBT (standard) Induction BCG or MMC (recommendation) Maintenance BCG or MMC (option)
High risk	TURBT Single, immediate postoperative chemotherapeutic instillation followed by: – BCG with a minimum of 1 year of maintenance (grade A) Immediate cystectomy may be considered for those at high risk of progression (grade C) and is recommended in patients with BCG failure (grade B)	TURBT (standard) Induction BCG with maintenance (recommendation) Cystectomy (option)

*Current (2012) EAU guidelines for NMIBC management [2] are similar to the 2008 EAU guideline recommendations*.

The use of clinical practice guidelines such as those described above can help minimise morbidity and improve the care of patients with NMIBC [8]. The Surveillance, Epidemiology, and End Results (SEER) study, for example, found a significant survival advantage among patients with NMIBC who received at least half of the guideline-recommended care for assessment and management [8]. However, reports suggest that many patients with NMIBC are not optimally managed as per current clinical practice guidelines [9–11].

The present individual patient data survey (IPDS) was designed to investigate clinical practice patterns in the management of intermediate- and high-risk NMIBC. In particular, the use of BCG in each of these risk categories was examined and compared with the 2008 (the year preceding the start of treating patients included in this IPDS) EAU and 2007 AUA guidelines for NMIBC management.

## Patients and Methods

Between 1 April 2011 and 30 April 2012, members of the IBCG randomly invited urologists in both community- and academic-based practices to participate in this retrospective on-line chart review. The only selection criterion for invitees was that these urologists treated patients with NMIBC in their respective practices.

Participating urologists were instructed to select the charts of the first 10 patients who underwent transurethral resection of bladder tumour (TURBT) in 2009 who met the following criteria: (i) patients had intermediate- (defined as multiple or recurrent low-grade tumours) or high-risk (defined as any T1 and/or high-grade/G3 tumours and/or CIS) NMIBC; and, (ii) patients had not been lost to follow-up. The 2009 period was chosen to ensure that at least 2 years of patient management and follow-up could be analysed.

For each chart, physicians completed an on-line survey consisting of seven questions related to diagnosis, planned treatment (including maintenance schedule used, number of instillations per course and duration of maintenance), treatment status (i.e. whether the planned treatment regimen was completed, discontinued or is currently on-going), and patient follow-up in the first, second, third and subsequent years after treatment (see Appendix for questionnaire). Frequency counts and associated percentages were used to analyse these variables as well as patient characteristics. The chi-squared or Fisher's exact tests were used to compare the practices of European vs North American physicians, and academic vs non-academic physicians. A *P* < 0.05 was considered to indicate statistical significance.

## Results

### Physician Participants and Patient Cohort

**Figure 1:**
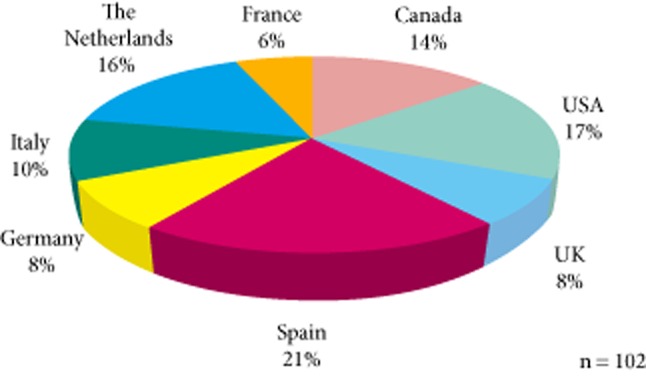
Breakdown of physician participants according to country.

In all, 300 urologists from North America and Europe were invited to take part in this retrospective on-line chart review; in all 102 (seven female, 95 male) participated (Fig. 1 shows the breakdown of physician participants according to country of practice); 53% were from academic institutions and 47% were based in non-academic practices.

In all, 1020 individual patient chart review surveys were completed; 49 were excluded due to missing or incomplete information. Therefore, 971 patients were included in the final analysis: 197 were classified as intermediate-risk and 774 as high-risk. Table 2 summarises the characteristics of the patient cohort.

**Table 2:** Characteristics of the patient cohort

Characteristic, n (%)	Risk level		Total (*n* = 971)
	Intermediate (*n* = 197)	High (*n* = 774)	
**Gender:**			
Male	148 (75.1)	624 (80.6)	772 (79.5)
Female	49 (24.9)	150 (19.4)	199 (20.5)
**Age category, years:**			
25–44	8 (4.1)	15 (1.9)	23 (2.4)
45–64	63 (32.0)	220 (28.4)	283 (29.1)
65–74	68 (34.5)	262 (33.9)	330 (34.0)
>74	58 (29.4%)	277 (35.8)	335 (34.5)
**Disease stage:**			
Ta	197 (100.0)	197 (25.4)	394 (40.6)
T1	0	577 (74.6)	577 (59.4)
**Tumour grade:**			
1973 WHO system:			
G1	56 (42.1)	24 (4.8)	80 (12.6)
G2	77 (57.9)	127 (25.2)	204 (32.0)
G3	0	353 (70.0)	353 (55.4)
2004 WHO system:			
Low-grade	144 (100%)	82 (13.4)	226 (29.9)
High-grade	0	530 (86.6)	530 (70.1)
**Presence of CIS**	0	225 (29.1)	225 (23.2)
**Number of tumours:**			
Single	32 (16.2)	377 (48.7)	409 (42.1)
2–7	150 (76.1)	370 (47.8)	520 (53.6)
>8	15 (7.6)	27 (3.5)	42 (4.3)
**Tumour diameter, cm:**			
<3	177 (89.9)	545 (70.4)	722 (74.4)
≥3	20 (10.1)	229 (29.6)	249 (25.6)
**Primary tumour**	63 (32.0)	524 (67.9)	587 (60.6)
**Recurrent tumour**	134 (68.0)	248 (32.1)	382 (39.4)

*High-risk defined as any T1, and/or high-grade/G3 and/or CIS; intermediate-risk defined as multiple or recurrent low-grade tumours (TaG1, TaG2)*.

### Treatment

Table 3 summarises the treatment of the most recent tumour in 2009 (whether primary or recurrent) of patients included in this analysis. In the intermediate-risk group, 46.7% of patients received EAU or AUA guideline-recommended adjuvant intravesical therapy: 29.4% received intravesical chemotherapy, 6.6% received BCG induction therapy and 10.7% received BCG induction plus maintenance. Of the high-risk patients, 50.1% received BCG induction plus maintenance as recommended by the EAU and AUA; 8.9% underwent cystectomy and 12.5% received intravesical chemotherapy. About 24% of intermediate-risk patients and 9% of high-risk patients received TURBT only, with no further intravesical or ‘other’ therapies.

**Table 3:** Treatment of intermediate- and high-risk patients

Treatment(s) used, n (%)	Risk level		Total (*n* = 971)
	Intermediate (*n* = 197)	High (*n* = 774)	
TURBT	**193 (98.0)**	**744 (96.1)**	937 (96.5)
Single immediate postoperative instillation of chemotherapy	**83 (42.1)**	266 (34.4)	349 (35.9)
Intravesical chemotherapy	**58 (29.4)**	97 (12.5)	155 (16.0)
BCG induction only	**13 (6.6)**	133 (17.2)	146 (15.0)
BCG induction + maintenance	**21 (10.7)**	**388 (50.1)**	409 (42.1)
Cystectomy	0	**69 (8.9)**	69 (7.1)
Other*	14 (7.1)	58 (7.5)	72 (7.4)
TURBT only (i.e. no intravesical therapy, ‘other’ therapies, or cystectomy)	48 (24.4)	70 (9.0)	118 (12.2)

*High-risk defined as any T1 and/or high-grade/G3 and/or CIS; intermediate-risk defined as multiple or recurrent low-grade tumours (TaG1, TaG2)*.

* *Other treatments included: surveillance, urethrectomy, radiotherapy, hormonal therapy, systemic chemotherapy, intravesical EMDA with MMC, nephroureterectomy, Synergo, outpatient laser fulguration, hyperthermic chemotherapy, celecoxib (BOXIT trial), interferon, and allopurinol (clinical trial)*.

*Note: bolded areas refer to EAU or AUA guideline-recommended therapy for the respective risk category*.

Table 4 compares the treatment of intermediate- and high-risk patients in Europe vs North America. Intermediate-risk patients in Europe were significantly more likely to receive a single, immediate postoperative instillation of chemotherapy (*P* = 0.03), intravesical chemotherapy (*P* < 0.001), and ‘other’ therapies (e.g. radiotherapy, systemic chemotherapy, intravesical electromotive drug administration (EMDA) with MMC, outpatient laser fulguration, hyperthermic chemotherapy, interferon; *P* = 0.04), while intermediate-risk patients in North America were more likely to receive BCG induction only (*P* < 0.001). High-risk patients in Europe were more likely to receive intravesical chemotherapy (*P* = 0.004), while high-risk North American patients were also more likely to receive BCG induction only (*P* < 0.001).

**Table 4:** Treatment of intermediate- and high-risk patients: Europe vs North America

Treatment(s) used, n (%)	Intermediate-risk			High-risk		
	Europe (*n* = 133)	North America (*n* = 64)	P	Europe (*n* = 551)	North America (*n* = 223)	*P*
TURBT	**130 (97.7)**	**63 (98.4)**	0.747	**527 (95.6)**	**217 (97.3)**	0.277
Single immediate postoperative instillation of chemotherapy	**63 (47.4)**	20 (31.25)	0.032	**193 (35.0)**	73 (32.7)	0.543
Intravesical chemotherapy	**53 (39.9)**	**5 (7.8)**	<0.001	81 (14.7)	16 (7.2)	0.004
BCG induction only	1 (0.75)	**12 (18.8)**	<0.001	78 (14.2)	55 (24.7)	<0.001
BCG induction + maintenance	**16 (12.0)**	**5 (7.8)**	0.369	**285 (51.7)**	**103 (46.2)**	0.163
Cystectomy	0	0	–	**46 (8.4)**	**23 (10.3)**	0.385
Other*	13 (9.8)	1 (1.6)	0.036	41 (7.4)	17 (7.6)	0.931

*High-risk defined as any T1 and/or high-grade/G3 and/or CIS; intermediate-risk defined as multiple or recurrent low-grade tumours (TaG1, TaG2)*.

* *Other treatments included: surveillance, urethrectomy, radiotherapy, hormonal therapy, systemic chemotherapy, intravesical EMDA with MMC, nephroureterectomy, Synergo, outpatient laser fulguration, hyperthermic chemotherapy, celecoxib (BOXIT trial), interferon, and allopurinol (clinical trial)*.

*Note: bolded areas refer to EAU or AUA guideline-recommended therapy for the respective risk category*.

There were some treatment differences between patients treated in academic vs non-academic-based practices. Intermediate-risk patients treated in academic practices were more likely to receive ‘other’ therapies *(*13%) than those treated in non-academic practices (2%; *P* = 0.002). High-risk patients treated in academic practices were less likely to receive BCG maintenance therapy (45% vs 55%; *P* = 0.009), and were more likely to undergo cystectomy (12% vs 5%; *P* < 0.001) or to receive intravesical chemotherapy (15% vs 9%; *P* = 0.01) than high-risk patients treated in non-academic practices.

### Maintenance BCG Regimens Used

Of the intermediate- and high-risk patients receiving BCG maintenance therapy (409 patients), most (93.1%) were scheduled to receive ≥1 year of maintenance as recommended by the EAU. Most patients were scheduled to receive at least three instillations per course (81.2%), and the most commonly used schedule was three weekly instillations at 3 months, 6 months and then every 6 months after BCG induction (51.8%; Table 5).

**Table 5:** Planned BCG maintenance schedule: duration, schedule of instillations, and instillations per course

BCG maintenance regimens used, n (%)	Risk level		Total (*n* = 409)
	Intermediate (*n* = 21)	High (*n* = 388)	
**Scheduled duration of maintenance, months:**			
<12	4 (19.1)	24 (6.2)	28 (6.9)
12	8 (38.1)	113 (29.1)	121 (29.6)
18	2 (9.5)	33 (8.5)	35 (8.6)
24	1 (4.8)	37 (9.5)	38 (9.3)
30	1 (4.8)	18 (4.6)	19 (4.7)
36	5 (23.8)	153 (39.4)	158 (38.6)
>36	0	10 (2.6)	10 (2.4)
**No. of BCG instillations per course:**			
1	1 (4.8)	61 (15.7)	62 (15.2)
2	0	15 (3.9)	15 (3.7)
3	16 (76.2)	233 (60.0)	249 (60.9)
>3	4 (19.0)	79 (20.4)	83 (20.3)
**Schedule of BCG maintenance instillations:**			
Monthly	3 (14.3)	36 (9.3)	39 (9.5)
Every 3 months	6 (28.6)	87 (22.4)	93 (22.7)
Every 6 months	2 (9.5)	32 (8.3)	34 (8.3)
At 3 months, 6 months, then every 6 months	8 (38.1)	204 (52.6)	212 (51.8)
Every 6–12 months or more	0	2 (0.5)	2 (0.5)
Only at recurrence	1 (4.8)	2 (0.5)	3 (0.7)
Other	1 (4.8)	25 (6.4)	26 (6.4)

### Treatment Status and Reasons for BCG Maintenance Discontinuation

In all, 37.2% of patients receiving BCG maintenance completed the maintenance regimen and 15.4% discontinued therapy; at the time of this analysis, the treatment regimen was on-going in 47.4% (Table 6). The most commonly cited reasons for discontinuation were: recurrence or progression (34.9%) and adverse events (34.9%) (Table 7); 80% of patients (16 of 20) who discontinued due to adverse events had cystitis (Table 8).

**Table 6:** Treatment status of patients scheduled to receive BCG maintenance

Treatment status, n (%)	Risk level		Total (*n* = 409)
	Intermediate (*n* = 21)	High (*n* = 388)	
Completed	10 (47.6)	142 (36.6)	152 (37.2)
Discontinued	1 (4.8)	62 (16.0)	63 (15.4)
On-going	10 (47.6)	184 (47.4)	194 (47.4)

**Table 7:** Reasons for BCG maintenance discontinuation

Reasons for discontinuation in patients who discontinued BCG maintenance therapy (*n* = 63)	N (%)
Adverse events/toxicity	22 (34.9)
Patient issues/concerns	7 (11.1)
Physician issues/concerns	2 (3.2)
Recurrence/progression	22 (34.9)
Death due to bladder cancer	1 (1.6)
Death due to other causes	6 (9.5)
Other	3 (4.8)

**Table 8:** Adverse events responsible for discontinuation of BCG maintenance therapy

Adverse event	N
Cystitis	5
Cystitis and fever	2
Cystitis and general malaise	2
Cystitis and haematuria	1
Cystitis and contracted bladder	2
Cystitis, fever and general malaise	2
Cystitis, contracted bladder and ureteral obstruction	1
Cystitis, fever, haematuria and granulomatous prostatitis	1
Contracted bladder and haematuria	1
Fever and general malaise	1
Epididymo-orchitis	1
Systemic BCG reaction/sepsis	1
Total	20

### Patient Follow-up

For most patients with high-risk NMIBC, follow-up or ‘planned’ follow-up was every 3 months for the first year (89.6%), every 3–6 months (94.9%) during the second year and every 6–12 months thereafter (82.7%). Follow-up or ‘planned’ follow-up for most intermediate-risk patients was every 3 months for the first year (80.2%), every 3–6 months during the second year (90.4%), and every 6–12 months thereafter (86.3%). Further information on patient follow-up will be the subject of a subsequent publication.

## Discussion

This retrospective chart review found marked underuse of guideline-recommended adjuvant intravesical therapy in patients with intermediate- and high-risk NMIBC. Only 50% of high-risk patients received BCG maintenance therapy as recommended by the EAU and AUA. Furthermore, intravesical chemotherapy was used in 12.5% of these patients, although it is not recommended for high-risk NMIBC. In intermediate-risk patients, only 47% received AUA or EAU guideline-recommended adjuvant intravesical therapy: BCG induction, BCG induction plus maintenance or chemotherapy. Although TURBT alone is not recommended for intermediate- or high-risk NMIBC, 24% of intermediate-risk patients and 9% of high-risk patients received only TURBT, with no further intravesical therapy. These findings are consistent with other studies that have found poor adherence to bladder cancer guidelines and underuse of BCG [9–11]. The American SEER study, which examined data from 685 patients diagnosed with primary NMIBC in 2003, found that intravesical therapy was used in only 31% of these patients. In the subset of 350 high-risk patients, only 42% received intravesical therapy [10]. Another recent study examining SEER-Medicare-linked data found that of the 4545 patients with high-grade NMIBC treated between 1992 and 2002, only one received all guideline-recommended measures for assessment and management. In addition, >40% of physicians had not performed at least one cystoscopy, one cytology, and one instillation of immunotherapy for a single patient nested within their practice during the initial 2-year period after diagnosis [9]. A study of 344 patients from eight Italian referral centres conducted in early 2009 found that BCG was used in only 66% of high-risk patients and, similar to the present study, intravesical chemotherapy was used as first-line therapy in 12.5% of these high-risk patients [11].

There were some differences in the treatment practices of European vs North American physicians and between physicians practicing in academic vs non-academic settings. High-risk patients in Europe were more likely to receive intravesical chemotherapy, while North American high-risk patients were more likely to receive BCG induction only. Intermediate-risk patients in Europe were more likely to receive a single, immediate postoperative instillation of chemotherapy and intravesical chemotherapy, and North American intermediate-risk patients were more likely to receive BCG induction only. These differences are probably due, at least in part, to the slight differences between the EAU and AUA guideline recommendations for NMIBC management. As mentioned earlier, the AUA guidelines recommend induction BCG for intermediate-risk NMIBC, which could explain the higher use of induction therapy in both intermediate- and high-risk North American patients. Also, the AUA guidelines recommend a single immediate chemotherapeutic instillation in low-risk patients, but not in intermediate- and high-risk patients that are to undergo further intravesical therapy. At the time of the present analysis, the EAU guidelines recommended a single immediate postoperative chemotherapeutic dose for all patients with NMIBC, which probably explains the higher use of a single immediate dose of chemotherapy in European intermediate-risk patients. Current EAU guidelines (2012) recommend a single-immediate postoperative chemotherapeutic dose as an option in high-risk patients who should go on to receive subsequent intravesical BCG immunotherapy [2].

Previous physician surveys have reported differences in NMIBC management practices depending on practice setting [12–14]. The present finding of greater use of cystectomy and ‘other’ therapies among patients treated by physicians in academic-based practices is expected, given that these physicians are probably affiliated with research, teaching or tertiary care centres that are experienced in the surgical aspects of bladder cancer management or that participate in clinical trials of novel bladder cancer therapies. The higher use of BCG maintenance therapy among non-academic physicians in the present study is similar to the results of a recent Bladder Cancer Advocacy Network survey of >500 urologists. This survey found the self-reported use of intravesical therapy to be higher among physicians in private practices vs academic-based practices (93% vs 85%; *P* = 0.01) [12].

Although several randomised clinical trials and meta-analyses have found that BCG with maintenance is superior to both chemotherapy and induction BCG alone for the prevention of recurrence and progression of intermediate- and high-risk NMIBC [15–23], some authors have questioned whether the routine use of maintenance BCG is justified, citing limitations in some of these past randomised trials and meta-analyses [24]. More recent findings from the EORTC 30911 trial comparing 3-week, 3-year maintenance BCG (with or without isoniazid) to maintenance epirubicin [21], the Japanese Cooperative Study comparing maintenance BCG to maintenance epirubicin [18] and an individual patient data meta-analysis of nine trials comparing MMC to BCG [22], confirm that better outcomes are obtained with BCG maintenance therapy compared with either induction therapy alone or intravesical chemotherapy. Although many patients will probably benefit from BCG maintenance therapy, the EORTC 30911 investigators have suggested that the use of maintenance should be considered on a case-by-case basis, taking into account the patient's previous history, his or her overall condition, and the patient's individual risk for recurrence and progression based on tumour characteristics [21].

Historically, BCG-associated adverse events were cited as common reasons for poor compliance to BCG therapy. However, with increasing experience in the use of BCG, side-effects are now less prominent, with <5% of patients having serious adverse events [25]. Furthermore, experts have emphasised that most adverse events can be managed effectively [25]. According to Lamm et al. [26], other patient- and physician-related factors are also responsible for non-adherence, e.g. patients' fears of side-effects and the treating urologist's personal opinions and beliefs about the value of BCG maintenance. In their analysis of SEER data, Chamie et al. [9] found that unexplained provider-level variation contributed significantly to the poor guideline adherence rate noted for cystoscopy (25%), cytology (59%), perioperative intravesical chemotherapy (45%), and postoperative instillations of BCG (26%). Recently, Lamm et al. [26] proposed various strategies for improving adherence to guideline recommendations for BCG maintenance use. These include: effective patient communication about the importance of BCG maintenance; increased knowledge about strategies for the management of adverse events; identifying potential barriers to non-adherence, e.g. lack of family support and concerns about side-effects; providing simple tools to help patients identify and track side-effects; and using a multidisciplinary team approach to monitor patients and reinforce the need for maintenance.

Despite the marked underuse of BCG noted in the present study, there appear to be some improvements in management as evidenced by the low maintenance discontinuation rate and the duration and schedule of maintenance instillations used. Previous studies have reported high rates of BCG maintenance discontinuation [19,27]. More recent EORTC trials of 3-week, 3-year BCG maintenance therapy have found discontinuation rates due to adverse events to range from 7% to 19% [21,28]. In the present study, 15% of patients scheduled to receive BCG maintenance discontinued maintenance therapy. Furthermore, of the 409 patients receiving BCG maintenance, only 22 (5%) discontinued due to adverse events. The low rate of maintenance discontinuation in the present study may be due, in part, to improvements in clinical practice and increased knowledge on the prevention and management of BCG-associated adverse events. In recent years, practical recommendations for the prevention and management of these adverse events have been published [25,29,30] and have emphasised that education on correct catheterisation techniques and the use of overall good clinical practice for BCG administration can help prevent side-effects in most cases.

Although the optimal schedule and dose of BCG has been the subject of debate over the last several years, the results of the Southwest Oncology Group (SWOG) 8507 study and the EORTC 30911 and 30962 trials suggest that the SWOG regimen of three weekly instillations at 3 and 6 months after induction and every 6 months thereafter for 3 years may lead to the best outcomes in patients with intermediate- and high-risk NMIBC [19,21,28]. The SWOG 8507 trial, for example, found recurrence-free survival (*P* < 0.001) and worsening-free survival (*P* < 0.04) to be significantly prolonged with 3-year maintenance BCG vs BCG induction only [19]. The EORTC 30911 trial found BCG maintenance (given as per the SWOG schedule) to be significantly better than maintenance epirubicin for prolonging time to first recurrence, time to distant metastases, and disease-specific and overall survival [21]. The EORTC 30962 trial randomly assigned 1355 patients with intermediate- and high-risk NMIBC to full-dose (81 mg) BCG for 1 year, one-third dose (27 mg) BCG for 1 year, one-third dose BCG for 3 years, or full-dose BCG for 3 years. BCG maintenance instillations were administered as per the SWOG schedule and the primary endpoint was the duration of the disease-free interval. Full-dose BCG for 3 years had the highest disease-free rate, while the one-third dose for 1 year had the lowest rate [28]. The AUA guidelines indicate that the best available evidence supports the use of the SWOG 3-week, 3-year maintenance regimen [6]. The EAU recommends at least 1 year of maintenance BCG and acknowledges that, based on the extent of intravesical immune response, three consecutive weekly instillations provide a maximum response [2]. In the present study, most patients (93%) who were treated with BCG maintenance were scheduled to receive ≥1 year of therapy and 39% were scheduled to receive 3 years of maintenance. In addition, a large proportion was treated according to the SWOG maintenance schedule (52%). Therefore, most physicians participating in this retrospective analysis appear to be using appropriate, evidence-based maintenance schedules.

Some limitations to the present study should be noted. It is a retrospective, non-randomised analysis of the medical records of patients treated in several different countries. The physicians who participated in the study were invited by members of the IBCG, thus creating an inherent selection bias. Urologists who agreed to participate may be more knowledgeable about and/or interested in the management of NMIBC than those who declined participation; therefore, poorer results may have been noted had a more formal randomised enrolment of urologists been performed. However, due to this ‘selection by association’ one could have expected greater adherence to guidelines than we found in the present study. Furthermore, as the findings of the present study are not based on a random sample of patients, it is unknown to what extent these results are representative of the overall population of intermediate- and high-risk patients with NMIBC.

Finally, a large proportion of patients included in this analysis were still undergoing treatment at the time of this analysis (47%) and the design of our survey does not allow for additional follow-up of these patients. Therefore, it is unknown whether these patients indeed completed the planned treatment regimen. However, given that evidence has shown that BCG is generally well-tolerated after induction and the first 6 months of maintenance therapy [27], it is likely that additional discontinuation in these patients will be low. Furthermore, the percentage of patients still undergoing treatment appears to coincide with the percentage of patients scheduled for >2 years of BCG maintenance therapy (46%), which suggests that these patients are tolerating BCG therapy.

Despite these limitations, we think that the present study highlights the under treatment of patients with intermediate- and high-risk NMIBC and underutilisation of BCG therapy, and further confirms the need for strategies to improve guideline compliance, particularly for the use of maintenance BCG in high-risk NMIBC. Future studies that identify barriers to adherence and strategies for improving provider-level adoption of guidelines for BCG use are critical to improving the care of patients with intermediate- and high-risk NMIBC.

## Abbreviations

CIS: carcinoma *in situ*
EAU: European Association of Urology
EMDA: electromotive drug administration
EORTC: European Organisation for Research and Treatment of Cancer
IBCG: International Bladder Cancer Group
IPDS: individual patient data survey
MMC: mitomycin C
NMIBC: non-muscle-invasive bladder cancer
SEER: Surveillance, Epidemiology, and End Results (study)
SWOG: Southwest Oncology Group
TURBT: transurethral resection of bladder tumour

## Supporting Information

Appendix International Patient Data Survey (IPDS).
