# Supplementary material for: Current clinical practice gaps in the treatment of intermediate- and high-risk non-muscleinvasive bladder cancer (NMIBC) with emphasis on the use of bacillus Calmette- Guérin (BCG): results of an international individual patient data survey (IPDS)
Source: BJU Int. 2013 Mar 1;112(6):742–50. doi: 10.1111/bju.12012 (PMC3933735; doi:10.1111/bju.12012)
Supplement: Appendix — International Patient Data Survey (IPDS). [file bju0112-0742-sd1.doc]

**Appendix: International Patient Data Survey (IPDS)**

1. **Patient Profile**
   - Gender: □ Male □ Female
   - Age: □ 25-44 □ 45-64 □ 65-74 □ >74
2. **Diagnosis at time of TURBT in 2009**

- Stage: □ Ta □ T1
- Grade (1973): □ G1 □ G2 □ G3 Grade (2004): □ Low-grade □ High-grade
- CIS: □ Yes □ No
- Number of tumours: □ Single □ 2-7 □ >8
- Tumour diameter: □ < 3 cm □ ≥ 3 cm
- Recurrence: □ Primary □ Recurrent *(if recurrent, answer 2b and 3b)*

**2b. What is the recurrence rate?**

- □ ≤ 1 recurrence/yr
- □ > 1 recurrence/yr

**3a. How was the primary tumour treated?**

- □ TURBT
- □ Single immediate dose of chemotherapy post TURBT
- □ Intravesical chemotherapy (go to question 4b)
- □ BCG induction only
- □ BCG induction + maintenance (go to question 4a)
- □ Cystectomy
- □ Other *(please specify):* ___________________
- Repeat TURBT: □ Yes □ No

**3b. How was the tumour that recurred in 2009 treated?** *(if recurrent tumour)*

- □ TURBT
- □ Single immediate dose of chemotherapy post TURBT
- □ Intravesical chemotherapy (go to question 4b)
- □ BCG induction only
- □ BCG induction + maintenance (go to question 4a)
- □ Cystectomy
- □ Other *(please specify):* ___________________
- Repeat TURBT: □ Yes □ No

**4a. Planned BCG maintenance schedule:**

- **Instillations per course:** □ 1 □ 2 □ 3 □ > 3
- **Duration of maintenance schedule:**
  - □ < 12 months
  - □ 12 months
  - □ 18 months
  - □ 24 months
  - □ 30 months
  - □ 36 months
  - □ > 36 months
- **Schedule of maintenance therapy instillations:**
  - □ Monthly
  - □ Every 3 months
  - □ Every 6 months
  - □ At 3 months, 6 months, then every 6 months
  - □ Every 6-12 months or more
  - □ Only at recurrence □ Other *(please specify): ___________________*

**4b. Planned duration of adjuvant chemotherapy:**

- □ < 3 months
- □ 3-6 months
- □ 6-9 months
- □ 9-12 months
- □ > 12 months

**5. What is the current treatment status?**

- □ Treatment plan completed (go to Question 7)
- □ Treatment plan ongoing (go to Question 7)
- □ Treatment plan discontinued (go to Question 6a)

**6a.** **Treatment plan discontinued:**

- **What was the primary reason for discontinuing the treatment plan?**
  - □ Adverse events/toxicity (go to Question 6b)
  - □ Patient issues/concerns (go to Question 6c)
  - □ Physician issues/concerns (go to Question 6d)
  - □ Recurrence/progression
  - □ Death due to bladder cancer
  - □ Death due to other causes
  - □ Other (please specify): _____(go to Question 7)

6b. **Please indicate the adverse event(s) responsible for treatment discontinuation:**

- □ Cystitis
- □ Hematuria
- □ Fever
- □ General malaise
- □ Contracted bladder
- □ Epididymo-orchitis
- □ Ureteral obstruction
- □ Granulomatous prostatitis
- □ Allergic/skin reactions
- □ Arthralgia
- □ Systemic BCG reaction/sepsis
- □ Other (please specify): __________________

6c. **Please indicate which of the following patient issues/concerns were responsible for treatment discontinuation:**

- □ Fear of potential adverse events
- □ Lack of knowledge on bladder cancer/therapy
- □ Costs
- □ Lack of caregiver/family, social or other support network
- □ Inconvenience of treatment protocol
- □ Lost to follow-up
- □ Other (please specify): ____________________

6d. **Please indicate which of the following physician issues/concerns were responsible for treatment discontinuation:**

- □ Cost/reimbursement issues
- □ Concerns regarding adverse events
- □ Concerns regarding systemic BCG reactions
- □ Uncertainty about the value of maintenance therapy
- □ Lack of facilities/resources
- □ Family physician relationship with patient
- □ Other (please specify): ____________________

**7.** **How frequently did you or do you plan to follow-up with this patient?**

- A) In the first year of treatment?

□ Every 3 months

□ Every 6 months

□ Annually

□ Other (please specify): ____________________

- B) In the second year of treatment?

□ Every 3 months

□ Every 6 months

□ Annually

□ Other (please specify): ____________________

- C) In the third and/or subsequent years of treatment?

□ Every 3 months

□ Every 6 months

□ Annually

□ Other (please specify): ____________________
